# Study of the Electrocatalytic Activity of Cerium Oxide and Gold-Studded Cerium Oxide Nanoparticles Using a Sonogel-Carbon Material as Supporting Electrode: Electroanalytical Study in Apple Juice for Babies

**DOI:** 10.3390/s130404979

**Published:** 2013-04-12

**Authors:** M. Yahia M. Abdelrahim, Stephen R. Benjamin, Laura Ma Cubillana-Aguilera, Ignacio Naranjo-Rodríguez, Josè L. Hidalgo-Hidalgo de Cisneros, Juan Josè Delgado, Josè Ma Palacios-Santander

**Affiliations:** 1 Departamento de Química Analítica, Facultad de Ciencias, Universidad de Cádiz, C/Republica Saharaui, S/N, 11510 Puerto Real, Cadiz, Spain; E-Mails: yahia_sci@yahoo.com (M.Y.M.A.); steaje@gmail.com (S.R.B.); laura.cubillana@uca.es (L.M.C.A.); ignacio.naranjo@uca.es (I.N.R.); jluis.hidalgo@uca.es (J.L.H.H.C.); 2 Departamento de Ciencia de los Materiales e Ingeniería Metalúrgica y Química Inorgánica, Facultad de Ciencias, Universidad de Cádiz, C/Republica Saharaui, S/N, 11510 Puerto Real, Cadiz, Spain; E-Mail: juanjose.delgado@uca.es

**Keywords:** cerium oxide nanoparticles, gold-studded cerium oxide nanoparticles, sonogel-carbon electrodes, ascorbic acid, structural characterization, electrochemistry, voltammetry, real sample, apple juice for babies

## Abstract

The present work reports a study of the electrocatalytic activity of CeO_2_ nanoparticles and gold sononanoparticles (AuSNPs)/CeO_2_ nanocomposite, deposited on the surface of a Sonogel-Carbon (SNGC) matrix used as supporting electrode and the application of the sensing devices built with them to the determination of ascorbic acid (AA) used as a benchmark analyte. Cyclic voltammetry (CV) and differential pulse voltammetry (DPV) were used to investigate the electrocatalytic behavior of CeO_2_- and AuSNPs/CeO_2_-modified SNGC electrodes, utilizing different concentrations of CeO_2_ nanoparticles and different AuSNPs:CeO_2_ w/w ratios. The best detection and quantification limits, obtained for CeO_2_ (10.0 mg·mL^−1^)- and AuSNPs/CeO_2_ (3.25% w/w)-modified SNGC electrodes, were 1.59 × 10^−6^ and 5.32 × 10^−6^ M, and 2.93 × 10^−6^ and 9.77 × 10^−6^ M, respectively, with reproducibility values of 5.78% and 6.24%, respectively, for a linear concentration range from 1.5 μM to 4.0 mM of AA. The electrochemical devices were tested for the determination of AA in commercial apple juice for babies. The results were compared with those obtained by applying high performance liquid chromatography (HPLC) as a reference method. Recovery errors below 5% were obtained in most cases, with standard deviations lower than 3% for all the modified SNGC electrodes. Bare, CeO_2_- and AuSNPs/CeO_2_-modified SNGC electrodes were structurally characterized using scanning electron microscopy (SEM) and energy dispersive X-ray spectroscopy (EDS). AuSNPs and AuSNPs/CeO_2_ nanocomposite were characterized by UV-vis spectroscopy and X-ray diffraction (XRD), and information about their size distribution and shape was obtained by transmission electron microscopy (TEM;. The advantages of employing CeO_2_ nanoparticles and AuSNPs/CeO_2_ nanocomposite in SNGC supporting material are also described. This research suggests that the modified electrode can be a very promising voltammetric sensor for the determination of electroactive species of interest in real samples.

## 1. Introduction

Due to their chemical and physical properties, nanomaterials, and in particular nanoparticles, are the subject of intensive research nowadays because of their scientific and technological importance. Specifically, gold nanoparticles (AuNPs) are employed in many fields: biosensors, cosmetics, nanoelectronic, catalysis, semiconductors, and biomedicine, among others [1–5]. This great attention and interest in AuNPs is due to their good biological compatibility, excellent conducting capability and high surface-to-volume ratio [6]. Recently, gold sononanoparticles (AuSNPs) have been obtained through a new, fast, cheap and green synthetic method using high power ultrasound under ambient conditions [7,8]. The sonosynthesis of AuSNPs is based on the decomposition in less than 6 min and with very low-energy costs of the precursor (potassium tetrachloroaurate, KAuCl_4_) in aqueous solution and its subsequent reduction and stabilization by a proper agent (sodium citrate trihydrate), all favored by irradiation with high power ultrasound. This kind of nanomaterial has proved to offer good and interesting electrocatalytic properties *versus* AuNPs produced by classical synthesis methods [9].

Oxide-based materials have emerged as alternative electrode surfaces in a number of electrochemical applications. Most of the studies deal with their use in electrochemical synthesis, electro-remediation and fuel cells [10]. On the contrary, electroanalytical applications have been relatively poorly investigated so far [11–14]. Besides, metal oxides have demonstrated to possess anti-fouling properties [15], what make them very useful for the determination of analytes in real samples. According to this, metal oxides might be very useful to modify electrochemical (bio)sensors in order to determine different kind of chemical species such as inorganic as organic ones. The studies reported in literature normally involve the use of Al_2_O_3_ [16]; other oxides, such as CeO_2_, WO_3_, TiO_2_ (rutile) and SnO_2_ [11,17], have been more rarely employed.

Nanostructured CeO_2_ is an excellent electrode material since it is a nontoxic, chemically inert and size-dependent electrically conductive material [18]. Many of its multiple applications are related to its oxygen transfer and storage ability: electrochemical redox couple for mediator-less glucose sensor and control of automotive emissions [19]; electrolyte material [20]; and promotion of CO oxidation in direct alcohol fuel cells by supplying oxygen ions to the Pt catalyst [21–23], among others. Electrochemical (bio)sensors based on CeO_2_-nanostructured-modified electrodes with enhanced electrocatalytic activity may facilitate the determination of many biomolecules. For example, some analytes determined by using CeO_2_-nanostructured-modified electrodes are: uric acid [24], ascorbic acid (AA) [25] and their mixture [12] at modified glassy carbon electrodes, dopamine [26] at a based carbon fiber microbiosensor, and urea [27] at an indium tin oxide (ITO)-coated glass substrate. However, their analytical applications are not much extended despite of the good selectivity, sensitivity, reproducibility and stability obtained for many of these devices, what make them promising voltammetric sensors for real sample analysis. The reason for this fact is usually attributed to the poor electrical conductivity of the electrodes based on this metal oxide, which limits their applications.

To overcome this shortage, the formation of nanocomposites, where at least one of the constituents possesses remarkable conductivity, may be a solution to increase sensitivity. In the literature it is possible to find many examples of these nanocomposites: Cu nanoparticles/ZnO [28], metal oxide/carbon nanotubes [29] and graphene/metal oxide core-shell nanostructures [30]. As far as we know there are only a few electroanalytical applications of similar CeO_2_-based nanostructures used as bi- or multi-functionalized electrocatalysts: TiO_2_/CeO_2_ nanoparticles [27] and Pd nanoparticles/CeO_2_ nanoparticles [31].

The present work reports the study of the electrocatalytic activity of CeO_2_ nanoparticles and AuSNPs/CeO_2_ nanocomposites. Both kinds of nanomaterials were deposited on the surface of a Sonogel-Carbon (SNGC) matrix by a simple drop-casting method and the sensing devices built with them were applied to the determination of AA, used as benchmark analyte. Different SNGC electrode configurations (CeO_2_ concentration and AuSNPs/CeO_2_ proportion used for the modification of the sensors) were tested. The advantages of employing CeO_2_ nanoparticles and AuSNPs/CeO_2_ nanocomposites in SNGC supporting material are also described. The electrochemical performance of the sensors was characterized by Cyclic Voltammetry (CV) and Differential Pulse Voltammetry (DPV). Up to the extent of our knowledge, this is one of the first electrochemical applications of AuSNPs/CeO_2_ nanocomposites as bi-functionalized electrocatalysts in (bio)sensors.

The electrochemical devices described in this paper were tested for the determination of AA in apple juice for babies, using High Performance Liquid Chromatography (HPLC) as a reference method. According to the type of real sample (apple juices for babies) in which the analyte is determined, the systems described in this work can be very promising tools for quality control purposes in food industry. It is noteworthy that this is the second time that exclusively electrochemical methods have been used to determine AA in commercial baby juices [9].

Finally, these devices were also structurally characterized by means of Scanning Electron Microscopy (SEM) and Energy Dispersive X-ray Spectroscopy (EDS). AuSNPs and AuSNPs/CeO_2_ nanocomposite were characterized by UV-vis spectroscopy, X-Ray Diffraction (XRD) and Transmission Electron Microscopy (TEM;.

## 2. Experimental Section

### 2.1. Reagents and Materials

Methyltrimethoxysilane (MTMOS) was from Merck (Darmstad, Germany) and HCl was from Panreac (Barcelona, Spain). KH_2_PO_4_ and K_2_HPO_4_ for phosphate buffer solution (PBS) and potassium hexacyanoferrate(II) were from Fluka (Buchs, Switzerland). Sodium citrate trihydrate was purchased from Scharlau (Scharlab, Barcelona, Spain) and potassium tetrachloroaurate(III), ascorbic acid and nanopowder cerium(IV) oxide (99.95%) form from Sigma-Aldrich (Sigma, Steinheim, Germany). Graphite powder natural, high purity-200 Mesh, 99.9999% (metal basis), was from Alfa-Aesar (Johnson Matthey GmbH, Germany). All reagents were of analytical grade and used as received without further purification. Nanopure water was obtained by passing twice-distilled water through a Milli-Q system (18 MΩ·cm, Millipore, Bedford, MA, USA). Glass capillary tubes, *i.d.* 1.15 mm (A = 0.0415 cm^2^), were used as the bodies of the composite electrodes. The nitrogen used for getting inert atmospheres and deaerating solutions in the measuring cell was N-55 type.

### 2.2. Instrumentation

The synthesis of the SNGC material was carried out by sonicating with a high power SONICATOR 3000 ultrasonic generator from MISONIX Inc. (Farmingdale, NY, USA) equipped with a 13-mm titanium tip, that provides a maximum output power of 600 W.

The voltammetric measurements were made on an Autolab® PGSTAT20 (Ecochemie, Utrecht, The Netherlands) potentiostat/galvanostat connected to a personal computer and a 663 Metrohm VA Stand module, using the software GPES (General Purpose Electrochemical System) 4.9 ver. for waveform generation, data acquisition and elaboration. The experiments were carried out in a single-compartment three-electrode cell, at room temperature (25 ± 1 °C). The counter electrode was a platinum wire, and a silver/silver chloride/3M KCl electrode was used as the reference. The composite-filled glass capillary tubes were used as the working electrode.

UV-Visible measurements were made with the Jasco 32 software using a Jasco V-550 (Easton, MD, USA) UV-visible spectrophotometer connected to a personal computer.

Scanning electron microscopy (SEM) studies were done on a QUANTA 200 (FEI Company, Hillsboro, OR, USA), normally operating at 20 keV and equipped with a Microanalyzer (EDAX) to perform energy dispersive spectroscopy (EDS). Both secondary electron and backscattered electron detectors were used to take the micrographs.

Transmission electron microscopy (TEM) studies were carried out on a JEOL JEM-2010F (Jeol, Tokyo, Japan) microscope, equipped with a field emission gun, a scanning-transmission electron (STEM) module, a high angle annular dark field detector (HAADF) and an energy dispersive X-ray spectroscopy (EDS) microanalyzer. The microscope was operated at 200 kV and in the STEM mode a 0.5 nm probe was used.

Finally, the X-ray diffraction pattern of dry CeO_2_ nanoparticles studded with AuSNPs at different w/w ratios, evaporated at 50 °C and supported on an oriented-silicon crystal with no background signal, was obtained using a Bruker D8 Advance X-ray diffractometer with Cu Kα radiation (λ = 0.1542 nm). Intensities were measured between 35° and 40° (2θ values) at intervals of 0.020°.

The results obtained by the electroanalytical techniques were compared with those obtained by HPLC, taken as a reference method. A JASCO HPLC system with UV detection and a Gemini C18 (250 mm × 3.0 mm) 5 μm particle size column was used to carry out the measurements. The analytical flow rate was 0.8 mL·min^−1^. As solvent a mixture of water−0.1% formic acid (v/v) was used. Detection wavelength was set at λ = 245 nm.

### 2.3. Sonogel-Carbon Electrode Preparation Procedure

To prepare the SNGC electrodes, the procedures described in literature were used [32]. Several minutes after beginning the gelification process of the sonosol, the capillary tubes were filled with the synthesized material to obtain the electrode as described elsewhere [33].

### 2.4. Electrochemical Pre-Treatment of Sonogel-Carbon Electrodes

Prior to the deposition of CeO_2_ or AuSNPs/CeO_2_ nanoparticles on the surface of the SNGC electrodes, they were electrochemically pre-treated by dipping them in 0.05 M sulphuric acid solution in the electrochemical cell, the SNGC electrodes operating as working electrode. The electrodes were polarized in CV from −0.5 to +1.5 V for 5 scans at a scan rate of 0.05 V (50 mV·s^−1^). Electrodes with similar current backgrounds were selected, carefully washed with Milli-Q water and dried at room temperature.

### 2.5. Synthesis of gold sononanoparticles (AuSNPs)

AuSNPs were synthesized as described in [7,8]. All the glass material used for the synthesis was cleaned with aqua regia (1:3 v/v HNO_3_-HCl) solution and then thoroughly rinsed with Milli-Q water prior to use. In general, gold nanoparticles show a characteristic surface plasmon band between 520 and 550 nm; hence, the formation of AuSNPs was monitored by using UV-visible spectrophotometry. After the synthesis, colloidal gold solution was stored in darkness conditions at 4 °C prior to use.

### 2.6. Preparation of CeO_2_ and AuSNPs/CeO_2_ Nanoparticle Solutions

On the one hand, several solutions of CeO_2_ nanoparticles at different concentrations (0.25, 0.5, 0.75, 1.0, 2.5, 5.0, 7.5 and 10.0 mg·mL^−1^) were prepared by dissolving a suitable amount of CeO_2_ nanoparticles in a certain volume of Milli-Q water.

On the other hand, six solutions mixing CeO_2_ nanoparticles and AuSNPs at different percentages (2.5%, 3.25%, 5%, 12.5%, 17.25% and 25% w/w AuSNPs:CeO_2_) were prepared by addition of the proper amount of CeO_2_ nanoparticles to the AuSNPs solution, prepared by ultrasonic synthesis, in order to obtain the desired proportions. Then, the six mixture solutions were stirred vigorously on a magnetic stirring hot-plate around 8 h. For TEM and DRX characterization, the solutions were centrifuged with a CENCOM 2 angular centrifuge to separate the CeO_2_ nanoparticles studded with AuSNPs, and afterwards, the precipitate was dried in a furnace at 50 °C for 24 h.

### 2.7. Preparation of the CeO_2_ and AuSNPs/CeO_2_ nanoparticle-modified SNGC sensors

CeO_2_ nanoparticles or AuSNPs/CeO_2_ nanocomposite solutions prepared as described above (3 μL), were deposited on the SNGC electrode surface and allowed to dry at room temperature in the darkness for 24 hours. After the drying, the electrodes were stored at 4 °C in darkness before and after their use. By following this process, the CeO_2_ and AuSNPs/CeO_2_ nanoparticles were well adhered to the surface of the electrodes.

### 2.8. Experimental Procedure

The experimental procedure can be described as follows: 25 mL of 0.2 M PBS buffer (supporting electrolyte, pH 6.9) were poured in the electrochemical cell placed in the Metrohm VA 663 Stand. After passing N_2_ for at least 15 min through the solution in order to deaerate it, the signal corresponding to the background was recorded three times, passing N_2_ for 1 min between two different sweeps. Next, the adequate amount of analyte was added into the cell to carry out the measurement step under the same conditions as the background.

The instrumental parameters for CV sweeps were as follows: potential range from −0.5 to +1.0 or −1.2 V; scan rates: from 5 to 200 mV·s^−1^. In the case of DPV, the optimal instrumental parameters were as follows: potential range from 0.5 to +0.7 V; modulation time: 0.05 s; interval time: 0.4 s; scan rate: 50 mV·s^−1^; step potential: 16 mV; and pulse amplitude: 100 mV.

In order to determine the concentration of ascorbic acid in real samples, the procedure was as follows: 1 mL of the commercial apple juice from local source (labeled concentration = 25 mg/100 mL= 250 mg·L^−1^ as average value) were diluted with phosphate buffer solution (0.2 M, pH = 6.90) until obtaining a final volume of 25 mL in an electrochemical cell, and then the electrode system was immersed into the solution. The electrochemical measurements were carried out under optimized DPV conditions, using the standard addition method.

Juice samples to be injected into the HPLC were prepared as follows: the real sample was diluted in sonicated Milli-Q water in 1:200 molar ratio. After measuring the real sample, different standard additions of AA in the range from 0.05 to 5 mg·L^−1^ with increments of 0.5 mg·L^−1^ each time were carried out.

## 3. Results and Discussion

### 3.1. Structural Characterization of AuSNPs and AuSNPs/CeO_2_ Nanoparticles

AuSNPs and AuSNPS/CeO_2_ nanoparticles were characterized by XRD and information about their size distribution and shape was obtained using the TEM technique.

#### 3.1.1. Transmission Electron Microscopy

**Figure 1.**
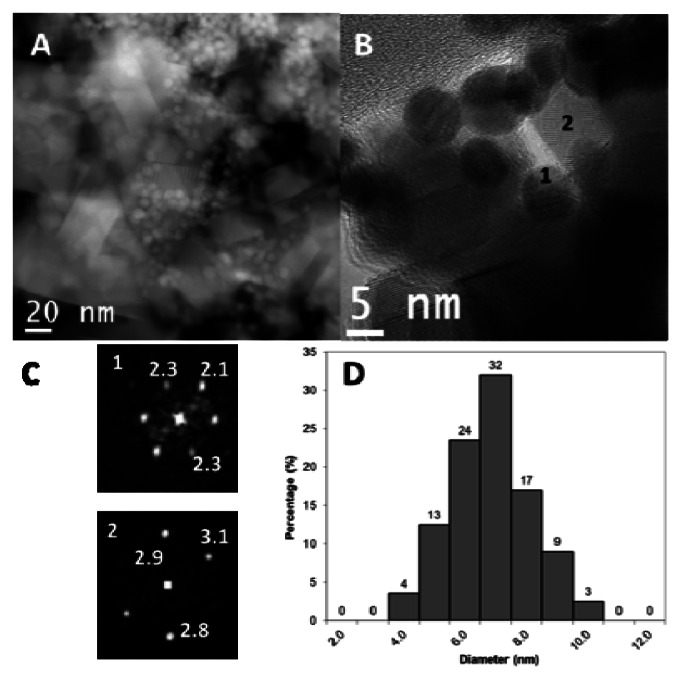
Typical HAADF-STEM (**A**) and HREM (**B**) micrograph of the sample. Digital Diffraction Pattern of selected areas (**C**) in image B and the particle size distribution (**D**) are included.

The morphology, shape and size distribution of AuSNPs were studied by TEM. As it can be clearly seen in Figure 1(A), AuSNPs are spherical-shaped and well dispersed from each other, with almost no aggregates (the rectangular and cubic forms correspond to CeO_2_ nanoparticles). Moreover, the size is very homogeneous following a Gaussian distribution, with a size distribution between 4 and 10 nm (Figure 1(D)). It should be pointed out that the particle size distribution was obtained using HAADF-STEM images in combination with elemental analysis by EDS to ensure a reliable particle size distribution. The 85% of the AuSNPs shows a diameter ranking from 5 to 8 nm. The average size is 7.1 ± 1.3 nm.

Regarding CeO_2_ nanoparticles studded with AuSNPs, Figure 1(A,B) show that the AuSNPs are completely distributed and dispersed over the CeO_2_ nanoparticles, which possess a heterogeneous morphology and an average size in the 30-50 nm range, in agreement with the reagent label. Besides, it seems that there is a good interaction between both kinds of nanoparticles. It must be taken into account that the AuSNPs/CeO_2_ nanoparticles were obtained by simply putting both nanomaterials in contact in solution, favored by the stirring process, with no calcination; hence, no more than physisorption-based interactions might be presumed between them, although the micrographs may point stronger links, even at the level of the crystalline structure itself. The deposition method employed to fix the sample to the grid was a simple drop-casting process, the same used for the deposition of this nanocomposite on the electrodes surface. The information retrieved from the Digital Diffraction Patterns (DDP) serve to corroborate the composition of the nanoparticles. As example, Figure 1(C) shows two typical DDP of a gold (1) and a ceria (2) nanoparticles, both of them recorded along the [011] zone axis in the [011].

#### 3.1.2. X-ray Diffraction

**Figure 2.**
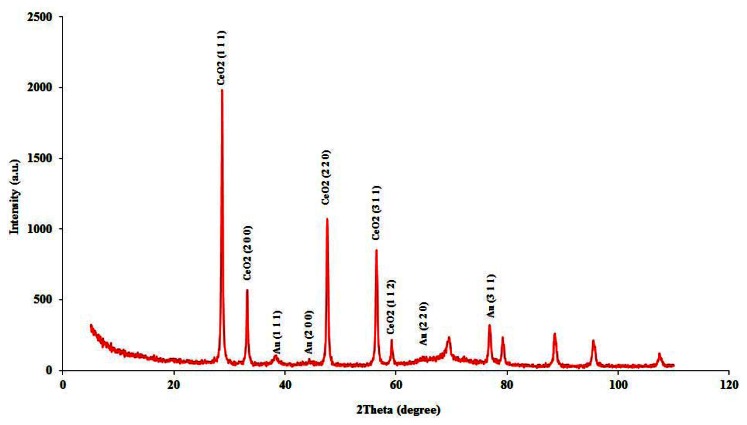
XRD pattern of AuSNPs/CeO_2_ nanoparticles.

The crystalline nature of AuSNPs and CeO_2_ was further confirmed from X-ray diffraction analysis. Because of the small size of the AuSNPs, the diffraction peaks were neither as intense nor as narrow as those observed for CeO_2_. As shown in Figure 2, the presence of four weak and broad peaks at 38.3°, 44.3°, 64.7° and 76.8° (2θ value) that can be indexed to the (1 1 1), (2 0 0), (2 2 0) and (3 1 1) planes, respectively corresponding to a face-centered cubic (fcc) phase of metallic gold, according to JCPDS No.04-0784. Concerning the CeO_2_ crystals, diffractogram shows three intense and narrow peaks at 28.6°, 47.6° and 56.4° (2θ value) that can be indexed to the (1 1 1), (2 2 0) and (3 1 1) planes, respectively corresponding to a cubic ceria fluorite (Fm3m) phase of CeO_2_, according to JCPDS No. 43-1002. Other less intense peaks can also be indexed for the same crystalline structure: 33.2° and 59.2° for (2 0 0) and (1 1 2) planes, respectively.

From the peak widths corresponding to CeO_2_, an average particle diameter, D̅, of 30.2 ± 3.0 nm was obtained according to Scherrer equation:

(1) $$D=\frac{0.9\cdot \lambda }{\beta \cdot \cos\theta }$$

where 0.9 is the shape factor, λ is the wavelength corresponding to the Cu Kα radiation (0.154060 nm), *θ* is the reflection angle, and *β* is the line broadening (peak width) at half the maximum. The results are coherent with the reagent label (D̄< 50 nm).

#### 3.1.3. UV-Visible Spectroscopy Studies

It is well established that UV-visible spectroscopy is a very effective technique to monitor the evolution of metal species in the synthesis of metal nanoparticles [34,35]. Thus, in our case, the formation of AuSNPs was followed by measuring, at room temperature, the absorbance of the ruby red color colloidal solution containing gold nanoparticles in the range 200-800 nm. The reduction of Au^3+^ to Au^0^ showed a maximum of absorption at 525 nm, indicating the production of the surface plasmon resonance effect, typical of the AuSNPs. Apart from being stable for more than one month, AuSNPs were also well dispersed in solution and with minimal aggregation [9].

UV-visible spectroscopy was also used to study the formation of the AuSNPs/CeO_2_ nanocomposite in solution at the six different w/w ratios. According to the Experimental Section, after 8 h of stirring, the different AuSNPs/CeO_2_ solutions were centrifuged. Later, the supernatants were collected and its absorbance spectra were recorded in the range 200-800 nm and at room temperature and at different stirring time, in order to know whether all the AuSNPs present in the original solutions were linked in some way to the CeO_2_ nanoparticles and what time of contact was necessary.

**Figure 3.**
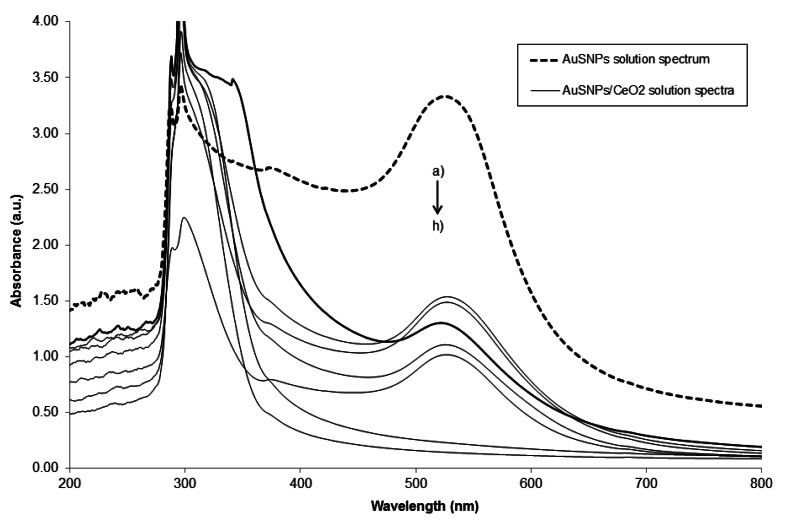
UV-visible spectra recorded for AuSNPs/CeO_2_ (5% w/w) nanoparticles at different stirring times: (**a**) AuSNPs spectrum immediately after the synthesis; AuSNPs/CeO_2_ nanoparticles spectra after stirring (**b**) 15 min; (**c**) 30 min; (**d**) 45 min; (**e**) 1 h; (**f**) 2 h; (**g**) 8 h and (**h**) 10 h.

Figure 3 shows several UV-visible absorption spectra for the AuSNPs/CeO_2_ nanoparticles at different stirring times. From the figure, we can conclude that the decoration of CeO_2_ nanoparticles with AuSNPs affects the absorption peaks of AuSNPs and it depends significantly on the stirring time. On one hand, it is obvious that an increase in the stirring time leads to an increased probability of contact between the AuSNPs and CeO_2_ nanoparticles; thus the ability to form the AuSNPs/CeO_2_ nanocomposite is enhanced. As it can be seen, when increasing the stirring time, the absorption bands of AuSNPs observed at 525 nm begin to decrease until they disappear completely after 8-10 h of stirring. That is why the value of 8 h was chosen as an appropriate contact time to prepare the AuSNPs/CeO_2_ nanocomposite solutions, since after this time no plasmon resonance absorption peak corresponding to free AuSNPs was observed. This selected value was also independent of the AuSNPs:CeO_2_ w/w proportion of the nanocomposite obtained.

### 3.2. Electrochemical Characterization

The electrochemical characterization of the unmodified, CeO_2_-modified and AuSNPs/CeO_2_-modified SNGC electrodes was conducted with two electroanalytical techniques: cyclic voltammetry (CV) and differential pulse voltammetry (DPV). In order to do that, fifteen electrodes with different configurations were tested: a bare or unmodified SNGC electrode, eight different CeO_2_-modified SNGC electrodes, with different concentrations of CeO_2_ nanoparticles (0.25, 0.5, 0.75, 1.0, 2.5, 5.0, 7.5 and 10.0 mg·mL^−1^), and six different AuSNPs/CeO_2_-modified SNGC electrodes, with different w/w proportions of AuSNPs:CeO_2_ nanocomposite (2.5%, 3.25%, 5%, 12.5%, 17.25% and 25%). The modified electrodes were built by depositing 3 μL of the corresponding suspension on the surface of previously pre-treated bare SNGC electrodes.

#### 3.2.1. Cyclic Voltammetry Studies

CV studies allow the evaluation of the electrochemical behavior of the devices (bare, CeO_2_-modified and AuSNPs/CeO_2_-modified SNGC electrodes) both in the presence and absence of 1.00 mM K_4_Fe(CN)_6_ solution. In presence of the mediator, the electrodes exhibited a pair of well-defined reversible redox peaks at ∼281 mV and ∼193 mV, which are attributed to the oxidation and reduction processes of iron in the mediator. The separation of the peak potential values: δE_p_ = E_a_−E_c_, is 88 mV. As it can be seen, this value is greater than 59/n (mV) as it should be for a totally reversible system. There was no redox peak in the absence of K_4_Fe(CN)_6_ (data not shown). It should be observed that the anodic and cathodic peak potentials did not shift when scan rates were increased, which can be also attributed to the reversible nature of the process. Stability of the electrodes has also proven to be rather good: after 20 successive measurements of K_4_Fe(CN)_6_ the results obtained for the peak current were almost the same, giving a relative standard deviation value less than 5%.

Regarding the electrochemical mechanism that takes place on the surface of the electrodes, when studying the relationship of anodic and cathodic peak currents as a function of the square root of the scan rate (*ν*^½^) (plots not shown), both parameters are proportional at the scan rates values studied (10-100 mV·s^−1^) with correlation coefficients greater than 0.99 for all the electrodes tested: e.g., *i*_pa_(μA) = 0.326 + 0.131·*ν*^1/2^(mV·s^−1^), *r* = 0.9982; and *i*_pc_(μA) = −0.262−0.138·*ν*^1/2^(mV·s^−1^), *r* = 0.9982 for the CeO_2_(10 mg·ml^-1^)-modified SNGC electrode, and *i*_pa_(μA) = 0.027 + 0.184·*ν*^1/2^(mV·s^−1^), *r* = 0.99997; and *i*_pc_(μA) = −0.035-0.186·*ν*^1/2^(mV·s^−1^), *r* = 0.9998, for the AuSNPs/CeO_2_(17.25% w/w)-modified SNGC electrode, which indicates diffusion-controlled kinetics towards the electrode surface [34–37]. More evidence for the non-adsorptive behavior of K_4_Fe(CN)_6_ was demonstrated when the sensor was subjected to cyclic voltammetry scans in 0.2 M PBS (pH 6.90). After being used in K_4_Fe(CN)_6_, no peak signal was obtained at all.

**Figure 4.**
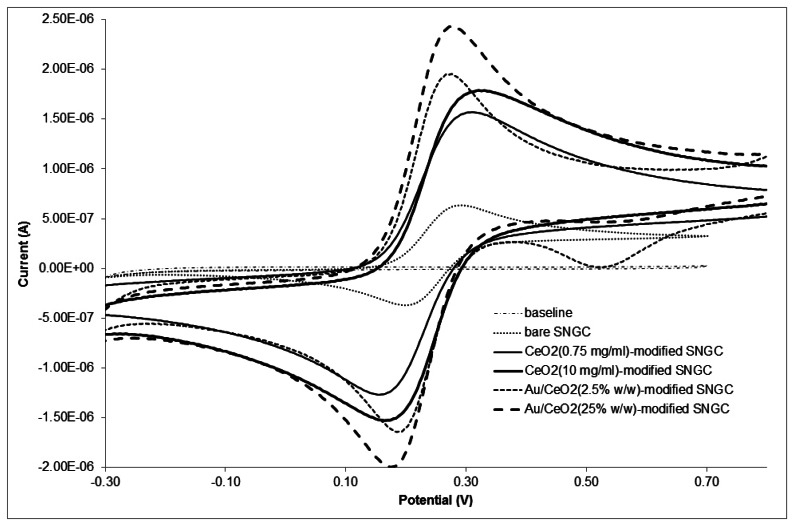
CV voltammograms corresponding to different configurations of the SNGC electrodes tested for 1.00 M of K_4_Fe(CN)_6_ recorded at 100 mV·s^−1^ of scan rate.

Figure 4 shows, as examples, the cyclic voltammograms corresponding to several electrodes: bare, CeO_2_(0.75 mg·L^−1^)-, CeO_2_(10 mg·mL^−1^)-, AuSNPs/CeO_2_(2.5% w/w)- and AuSNPs/CeO_2_(25% w/w)-modified SNGC electrodes both in presence and absence of 1.00 mM K_4_Fe(CN)_6_ solution at 100 mV·s^−1^. As it can be seen, the peak intensity values for both anodic and cathodic peaks are higher for the SNGC electrode modified with CeO_2_ nanoparticles or AuSNPs/CeO_2_ nanocomposite than for the bare SNGC electrode. This is due to the more electroactive surface of the CeO_2_- and the AuSNPs/CeO_2_-modified SNGC electrodes compared to the unmodified one. The presence of CeO_2_ nanoparticles and AuSNPs/CeO_2_ nanocomposite on the electrodes surface increases the superficial area, and thus the electrochemical response versus the mediator [K_4_Fe(CN)_6_]. Furthermore, the peak intensity for the CeO_2_ (10 mg·mL^−1^)-modified SNGC electrode was higher than that for the same type of electrode but with less CeO_2_ nanoparticles concentration, as shown in the figure. This is obviously due to an increase in the concentration of CeO_2_ nanoparticles deposited on the electrode surface which leads in turn to an increase in the number of CeO_2_ nanoparticles at the electrode surface, increasing the number of electroactive sites, and hence the peak intensity as well.

Besides, the peak intensity for AuSNPs/CeO_2_ (25% w/w)-modified SNGC electrode was the highest, as Figure 4 shows. This fact can be explained in terms of the amount of CeO_2_ nanoparticles deposited on the electrodes' surface. Since the AuSNPs/CeO_2_ (25% w/w)-modified SNGC electrode presented the lowest amount of CeO_2_ nanoparticles of all the electrochemical devices built, and CeO_2_ nanoparticles possess greater isolating character than AuSNPs, the number of electroactive sites of AuSNPs increases at the electrode surface, and hence the peak intensity and the electrochemical response versus the mediator [K_4_Fe(CN)_6_] as well. The same conclusions could be reached by taking into consideration the amount of gold on the devices' surface: the higher the AuSNPs:CeO_2_ proportion, the higher the current intensity. This result might suggest that electrodes modified with AuSNPs/CeO_2_ nanocomposites perform better than electrodes modified only with CeO_2_ nanoparticles, due to its isolating properties. Moreover, SNGC electrodes modified with the AuSNPs/CeO_2_ nanocomposite allow a shift in the mediator anodic peak towards less positive potentials: from ∼0.310 V (corresponding to the maximum of the peak for CeO_2_-modified electrodes) to ∼0.266 V (electrodes with the nanocomposite). However, antifouling properties of metal oxides should also be taken into account when considering the electrochemical performance of the devices.

**Figure 5.**
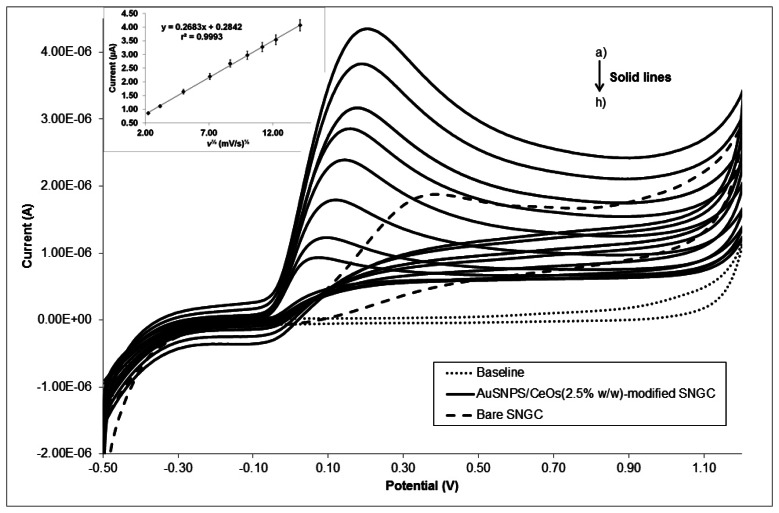
CV voltammograms of 1.0 mM AA, in 0.10 M PBS (pH 6.9) at AuSNPs/CeO_2_ (2.5% w/w)-modified SNGC electrode (solid lines) at different scan rates: (**a**) 5; (**b**) 10; (**c**) 25; (**d**) 50; (**e**) 75; (**f**) 100; (**g**) 150; (**h**) 200 mVmiddot;s^−1^), and at bare SNGC electrode (dashed line) at 50 mV middot;s^−1^. The inset displays the plot of peak current against square root of scan rate.

Figure 5 depicts, as an example, the CV responses of AA at AuSNPs/CeO_2_ (2.5% w/w)-modified SNGC electrode (solid lines) under various scan rates, as well as the bare SNGC response (dashed line) versus this mediator at 50 mV s^−1^. The plot of the redox peak current as function of the square root of scan rates is shown in the inset.

As it can be seen, good linear relationship is observed, indicating that this compound is electroactive under diffusion-controlled process at the AuSNPs/CeO_2_(2.5% w/w)-modified SNGC electrode. This figure also shows that AA is irreversibly electro-oxidized at the sensing device.

In order to get the information on the rate-determining step, Tafel slope, *b*, was determined using the following equation valid for a totally irreversible diffusion-controlled process [38]:

(2) $$E_{p}=\left(\frac{b}{2}\right)\log\upsilon +\text{constant}$$

Therefore, on the basis of Equation (2), the slope of *E*_p_ versus log *υ* plot is:

(3) $$\frac{\text{d}E_{p}}{d\log\upsilon }=\frac{b}{2}$$

where *b* is the Tafel slope and *υ* is the scan rate; the Tafel slope can also be expressed as:

(4) $$\text{b}=2.3\text{RT}{\left(\alpha_{a}n_{\alpha }F\right)}^{-1}$$

On the basis of these equations, the slope of the plots of *E*_p_ versus log *υ* is *b*/2 which was found equal to 0.0628 in this work as the following linear equation states: *E*_p_ (V) = 0.0628·log *υ* (mV/s) + 0.0275, with a *r*^2^ value equal to 0.9992; so, *b* = 2×0.0628 *V* = 0.126 V. It is known that ascorbic acid oxidation kinetics on many materials [38–41] occurs with single electron transfer process. Assuming this, these slope values indicate a transfer coefficient (α) equal to 0.4699, which in most systems turns out to lie between 0.3–0.7 [35].

**Figure 6.**
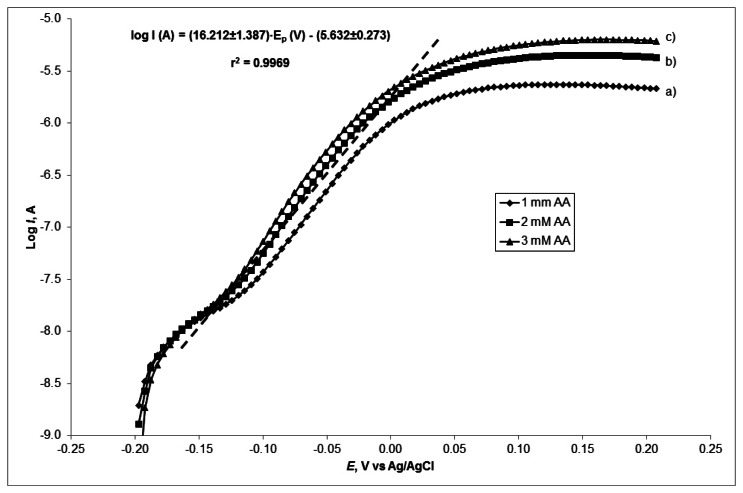
Tafel plots derived from current-potential curves obtained on the AuSNPs/CeO_2_(2.5% w/w)-modified SNGC electrode in the presence of different AA concentrations: (**a**) 1, (**b**) 2 and (**c**) 3 mM at a scan rate of 50 mV·s^−1^ in PBS 0.2 M.

The number of electrons involved in the rate determining step, can be obtained using another method. Tafel plots (Figure 6) were drawn using the data from the rising part of the current-voltage curve at a scan rate of 50 mV·s^−1^ for three different concentration of AA: 1, 2 and 3 mM.

A mean slope of 0.0617 V·decade^−1^ or 16.21 (V·decade^−1^)^−1^ was obtained presenting a one-electron process which was rate limiting assuming a transfer coefficient of α = 0.4785. The results obtained from the two different methods are in good agreement.

Some questions concerning the capacity of the electrodes should be stated. This parameter gives an idea about the amount of charge that is stored in the electrode at a given potential value. It corresponds to the non-faradaic current, *i.e.*, the amount of charge that is not used to oxidize or reduce an electroactive species. To evaluate the behavior of our electrodes, cyclic voltammograms were carried out in phosphate buffer solution 0.2 M at pH 6.9. In our study we have calculated the corresponding values of the experimental observed capacity and the double-layer capacity (Table 1). The observed capacity is defined as *C*_obs_ = *i*/*ν*, where *i* is the average anodic and cathodic current density and *ν* is the scan rate (100 mV·s^−1^), and the double-layer capacity (*C*_dl_) consists of the slope of the regression line obtained when representing the average (absolute) values of the anodic and cathodic current densities at different scan rates (from 10 to 100 mV·s^−1^) versus the scan rate values. As an example, we show the regression equation: *J*_average_ (μA·cm^−2^) = 149.656·*ν* (mV·s^−1^) + 0.333, *r* = 0.9988, corresponding to the CeO_2_(0.5 mg·ml^−1^)-modified SNGC electrode.

As observed in Table 1, as expected the lower value for the capacities corresponds to the unmodified SNGC electrode, taken as reference value, since this parameter increases its values because of the presence of chemical species (modifiers) on the surface of the electrodes. The *C*_obs_ value for the unmodified electrode shows perfect correspondence with previously published results [32,42]. On one hand and in general terms, when depositing CeO_2_ nanoparticles on the surface of the SNGC electrodes the capacity values increase: the higher the amount of nanoparticles, the greater the capacity values. This is probably due to the resistive character of the CeO_2_ film. On the other hand, concerning the AuSNPs/CeO_2_-modified SNGC electrodes, the capacity values decreases with the AuSNPs/CeO_2_ percentage (consider that the lower the percentage value, the higher the CeO_2_ concentration), despite of the presence of AuSNPs. This might be attributed to the fact that gold nanoparticles are not directly in contact with the electrode surface and that current mainly flows through the CeO_2_ nanoparticles (in a very high percentage with respect to gold) to the transducer. According to this, it seems that when modifying SNGC electrodes with the AuSNPs/CeO_2_ nanocomposite, the capacity values are lower than when modifying only with CeO_2_. This means that a AuSNPs/CeO_2_-modified SNGC electrode stores much less amount of charge, and hence much more charge is available to oxidize and/or reduce the analytes. In general, the lower the capacity values, the better the electrochemical performance of the electrodes.

**Figure 7.**
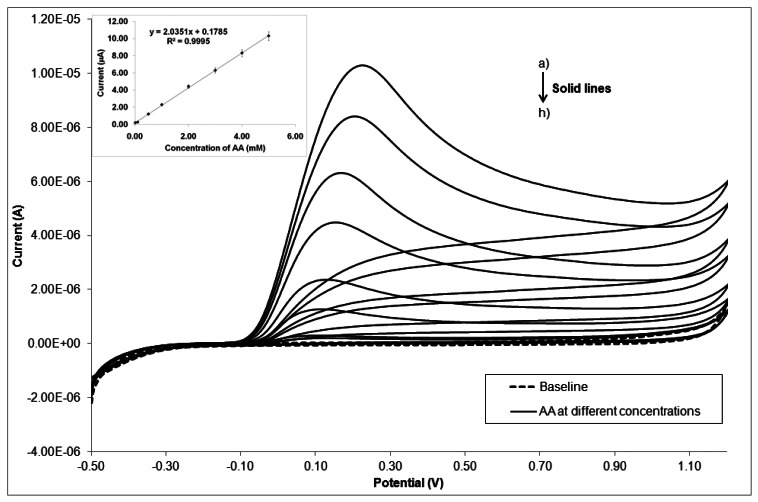
The cyclic voltammograms of AuSNPs/CeO_2_(2.5% w/w)-modified SNGC electrode in 0.2 M PBS with different concentrations of ascorbic acid: (**a**) 0.01; (**b**) 0.1; (**c**) 0.5; (**d**) 1.0; (**e**) 2.0; (**f**) 3.0; (**g**) 4,0; (**h**) 5,0 mM. The inset displays the AA oxidation peak current on this electrode versus concentration of the analyte.

To finish with the CV studies, the effect of ascorbic acid concentration on the cyclic voltammetric response of CeO_2_- and AuSNPs/CeO_2_-modified SNGC electrodes was investigated. Figure 7 shows, as an example, the cyclic voltammograms of the AuSNPs/CeO_2_(2.5% w/w)-modified SNGC electrode at the presence of various concentrations of ascorbic acid. With increasing AA concentration in the solution, the anodic peak current is increased. This catalytic peak current has a linear relationship with the concentration of AA in the range of 0.01–5 mM with a correlation coefficient of 0.9995 (inset of Figure 7). From these results, we can conclude that the electro-oxidation of AA on these electrodes can be used for the quantitative determination of this analyte in samples, being the simplicity of preparation of the electrodes described here other advantage to be taken into account.

#### 3.2.2. Differential Pulse Voltammetry Studies

Pulse voltammetry techniques have been established to be very sensitive in the detection of micromolar amounts of chemical species, particularly AA [9,34,43]. Thus, differential pulse voltammetry (DPV) has been used to evaluate and demonstrate the good electrochemical performance of our devices when determining AA selected as benchmark analyte.

In the literature, the effect of the DPV parameters on the response of the electrodes has been studied by means of three factors and three levels Box-Behnken experimental design [44]. These investigations allowed us to individuate the scan parameters leading to optimal sensitivity of the devices. According to our experience and previous studies [9], the optimal combination of DPV parameters chosen for these studies was as follows: interval time (IT) = 0.4 s, step potential (SP) = 16 mV and modulation amplitude (MA) = 100 mV. Using this combination of parameters several calibration curves for AA at the scan rate of 50 mV·s^−1^ were obtained by using the SNGC electrodes at different configurations. The potential range at which the sensor responses were studied was from −0.5 V to 0.7 V in a 0.2 M PBS (pH 6.9) buffer solution.

**Figure 8.**
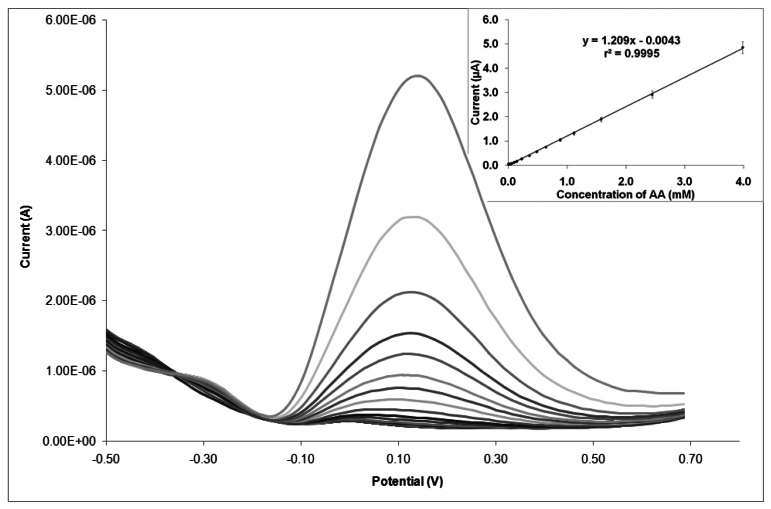
DPV voltammograms corresponding to one calibration curve (inset) for ascorbic acid using a AuSNPs/CeO_2_ (5% w/w)-modified SNGC electrode.

Together with the determination coefficient that give us an idea about the goodness of the regression model, special attention was also paid to the slope of the regression lines, which represents the sensitivity (measured in A·M^−1^) of the sensor for this analyte. The calibration curves were obtained for an AA concentration range from 1.5 × 10^−6^ M (0.25 mg·L^−1^) to 4.0 × 10^−3^ M (700 mg·L^−1^). For each calibration curve, 18 points at different AA concentrations within this range (three orders of magnitude) were measured, with three replicates. One example of the calibration curve obtained for AA when using AuSNPs/CeO_2_(5% w/w)-modified SNGC electrode is: *I*(A) = 1.209 × 10^−3^· [AA] (A·M^−1^)–4.265 × 10^−9^, *r^2^* = 0.9995. The relationship between concentration of AA and current peak height was linear in the concentration range studied. In Figure 8, an example of the DPV voltammograms corresponding to the calibration curve (inset) for electrocatalysis of ascorbic acid is shown.

Table 2 shows the calibration curves obtained for the different types of SNGC electrodes used throughout this research work, in terms of slope and determination coefficient values. From the table, it can be seen that the electrochemical performance of electrodes modified with CeO_2_ and AuSNPs/CeO_2_ nanoparticles are similar. On one hand, it seems that in general the higher the CeO_2_ concentration on the electrode surface, the lower the slope of the calibration curves and hence the sensitivity. This can be explained in terms of the resistive character of the CeO_2_ film: the current must flow through this film to the electrode surface. On the other hand, regarding the AuSNPs/CeO_2_-modified-SNGC electrodes, similar results could be obtained: electrodes modified with higher proportion of AuSNPs seem to offer better sensitivity (consider that the higher the percentage, the lower the CeO_2_ concentration). However, it seems that the decoration with AuSNPs does not show significant advantages with respect to sensitivity, perhaps because of the high thickness of the CeO_2_ film, which seems to be a determinant factor concerning sensitivity of the electrodic devices. The modified device that shows the higher sensitivity corresponds to AuSNPs/CeO_2_ (25% w/w)-modified SNGC electrode, which is also one of the electrochemical devices with the lowest capacity values when measuring K_4_Fe(CN)_6_. For application purposes, it would be advisable to achieve a compromise between sensitivity and capacity.

When comparing bare and modified SNGC electrodes; the sensitivity is higher with no modifications of the electrode surface. This is probably related to the place where electrochemical reactions (redox reactions) of the analyte takes place; from the results obtained; it seems that electrooxidation and/or electroreduction occurs at the surface of the bare SNGC electrode; but in case of modified-SNGC devices these reactions occur at the surface of CeO_2_ and AuSNPs/CeO_2_ nanoparticles. The presence of the CeO_2_ nanoparticles film at the surface of the electrodes increases their electrochemical resistance. Moreover; due to the different nature of the electrocatalytic processes taking place on smooth electrode surface and on nanoparticles surface; it might not be possible to compare directly the slopes of bare and modified SNGC electrodes (or at least doing it with special care).

Reproducibility and repeatability studies were performed as well. In the case of the reproducibility studies, several calibration curves for AA were obtained by using three different SNGC electrodes for each one of the configurations tested. The results expressed in terms of the average slope and the respective coefficients of variation are shown in Table 3.

As it can be seen, the results are coherent with those reported previously in Table 1. The trend and the average slope values, in general, are similar for all the configurations, excepting for the bare SNGC electrode, due to the same reasons discussed previously. Regarding the coefficient of variation for the slope, excepting for the less concentrated CeO_2_-modified devices and the one deposited with AuSNPs/CeO_2_(25% w/w), the values are rather good in general, being better for AuSNPs/CeO_2_- than for CeO_2_-modified SNGC electrodes and remarkable for CeO_2_(2.5 mg·mL^−1^)-, AuSNPs/CeO_2_ (5% w/w)- and AuSNPs/CeO_2_ (17.25% w/w)-modified SNGC. This means that: (i) the three quoted electrodes perform better in terms of reproducibility, despite the high number of measurement that are necessary to be done with only one electrode to obtain a single calibration curve; (ii) the process of fabrication of the electrodes is also very reproducible, what is very difficult for modified composite electrodes (the Sonogel-Carbon material fabrication process and the deposition of CeO_2_ and AuSNPs/CeO_2_ nanoparticles); iii) for application purposes, a compromise among reproducibility, sensitivity and capacity values should be achieved.

With respect to the repeatability studies also reported in Table 3, a SNGC electrode for each configuration was tested and the average slope and the coefficient of variation for the slope for at least three calibration curves using the same electrode were also obtained. In general, modified SNGC electrodes showed similar and rather good repeatability results. Moreover, excepting for several AuSNPs/CeO_2_-modified SNGC electrodes, the repeatability values were remarkable good in all cases (under 7%–8%). It seems that for devices with higher coefficient of variation value some problem with the deposition took place or an unexpected degradation of the electrode surface (see Section 3.4.1) occurred due to the weight and thickness of the film as well as the intensive use of the amperometric sensor.

Some comments concerning the detection and quantification limits (LD and LQ, respectively) for the electrodes tested at different configurations are warranted. Table 3 shows the values obtained for both LD and LQ for each configuration of the SNGC electrodes. LD was calculated as three times the standard deviation of the blank and LQ as ten times this value as reported by Miller and Miller [45]. On one hand, in case of CeO_2_-modified SNGC electrodes, the lower the concentration of CeO_2_ nanoparticles, the better the LD and LQ values, being remarkable for the CeO_2_(0.75 mg·ml^−1^)-modified SNGC electrode; however, it should be taken into accoSunt that reproducibility of this electrode is not very good. Perhaps CeO_2_(10.0 mg·mL^−1^)-modified device could be considered instead. Nevertheless, these values are more or less similar in all cases and within the units or tenths of micromolar range. On the other hand, concerning, AuSNPs/CeO_2_-modification, the lowest LD and LQ values were obtained for AuSNPs/CeO_2_(3.25% w/w)-modified SNGC electrode, although as it happened with the CeO_2_ modified devices, the results were similar in all cases. Finally, when comparing between bare and modified-SNGC electrodes, the best values are clearly obtained for modified ones. This means that both CeO_2_ and AuSNPs/CeO_2_ nanoparticles, overall the first one for its resistive character and its higher concentration versus AuSNPs, play an important role in the determination of AA.

In comparison with some reported AA sensors [9,12,31,41,46–57] (see Table 4), modified with CeO_2_ nanoparticles, AuNPs, AuNPs/CeO_2_ nanocomposite, or with other types of modifiers, the electrochemical device that we propose shows very important advantages with respect to others found in literature: (i) SNGC electrodes are modified with both types of nanomaterials (AuSNPs and CeO_2_ nanoparticles) simply by drop casting method, and the proposed methodology shows results that are in the same range, and in some cases, are better than most of those reported in literature by using more complex systems for electrode modification; (ii) some of the CeO_2_- and AuSNPs/CeO_2_-modified SNGC electrodes are rather sensitive with a relatively high linear range simultaneously; besides, these modified electrodes are easy of fabrication, low cost and can be used for many times, showing high reproducibility and repeatability; (iii) the electro-oxidation signal corresponding to AA appears at less positive potentials values (0.140 V) than most of methodologies reported in literature. This research suggests that these modified electrodes could be very promising voltammetric sensors for the determination of AA. It has to be taken into consideration that there are only a few papers (see Table 4) reporting determination of AA with CeO_2_-modified electrodes and that up to the extent of our knowledge this is the first time that AA has been determined by means of a AuNPs/CeO_2_ nanocomposite-modified electrode.

Moreover, when comparing our system with other electrochemical quantification methods for AA, it is noteworthy that DPV is the most extended method to determine AA. According to Table 4, amperometry leads to lower detection limits than typical voltammetric techniques as CV or DPV, and when using SWV results are rather good, being in a similar range than those obtained for DPV. However, when looking at DPV only, it seems that the improvement in the figure of merits depends strongly on the electrochemical device employed, and mainly in the modification of the electrode. Our devices, modified with CeO_2_ nanoparticles and AuSNPs/CeO_2_ nanocomposite, as stated before, performs rather well, with lower detection limits and greater dynamic range than other more complex modified devices such as β-CD-nanoAu/Fc-ITO, AuNPs/silica/MWCNT/GCE or AuNPs/β-CD/graphene/GCE. Besides, some other advantages, as those described above, can be noted as well.

### 3.3. Real Sample Application

Some tests of the electrochemical devices used throughout this work were done to determine ascorbic acid (vitamin C) in commercial apple juices for babies. The determination of AA in the real samples was carried out by the standard addition method in order to prevent any matrix effects. Moreover, the results obtained by the electroanalytical techniques were compared with those obtained by HPLC, taken as a reference method. It is noteworthy that this is the first time that electrochemical sensors based on the nanocomposite as the one reported in this work has been applied to the determination of AA in commercial apple juices for babies as real sample.

Table 5 shows the experimental results obtained for AA in the real sample measurements. The best recovery percentage values, calculated from three replicates, are shown in the table, as well as the configuration of the electrodes used for the measurements. According to the reference method employed, the concentration of AA is 1.24 × 10^−3^ M (218.0 mg·L^−1^) (the label of the real sample was 1.42 × 10^−3^ M (250.0 mg·L^−1^) as an average value).

According to Table 5, in general, the results are remarkable and very similar in all cases. The error in the recovery percentage only goes beyond 5% in a few cases. After analyzing the recovery values, it can be concluded that CeO_2_- and AuSNPs/CeO_2_-modified SNGC electrodes give percentage values slightly higher and lower than 100%, respectively. However, in general, the results obtained are rather good, with relative standard deviation (RSD) less than 6% in all cases, and precision (p-value = 0.05) lower than 5%. Besides, the AuSNPs/CeO_2_-modified SNGC electrodes show lower standard deviation values in accordance to reproducibility and repeatability studies. According to scientific literature and as far as we know, this is the second time that the electrochemical determination of AA in baby juices is reported [9]. Other methods have been usually used: enzymatic, fluorimetric, chromatographic, but not exclusively electrochemical ones.

### 3.4. Structural Characterization of CeO_2_- and AuSNPs/CeO_2_-Modified SNGC Electrodes

The structural characterization of the surface of the SNGC electrodes at the different configurations was carried out by employing several instrumental techniques: SEM and EDS. As in the electrochemical studies, the configurations tested for the electrodes were: one bare, eight different concentrations of CeO_2_-modified and six different proportions of AuSNPs/CeO_2_-modified SNGC electrodes. For each sample the corresponding electrode used and the electrode not used were analyzed.

#### 3.4.1. Scanning Electron Microscopy and Energy Dispersive X-ray Spectroscopy

For each sample, the SEM and EDS studies were performed on the same equipment and at the same time, as it was mentioned in the experimental section. As SEM studies were carried out at low vacuum, it was not necessary a previous step of coating the samples with gold. The micrographs were taken at 20 kV in all cases and at different magnifications: 90×, 160×, 300× and 600×. Higher magnifications were used to observe some interesting details, mainly when studying the presence of CeO_2_ and AuSNPs on the surface of the electrodes. The main conclusions that can be obtained after applying this technique are summarized as follows:

1. The use of the voltammetric device, either bare or modified, causes small erosion of the electrode surface in the form of holes and fissures, and a greater separation between the composite material and the wall of the glass-capillary tubes. This erosion is mainly produced during the electrochemical polarization or activation of the SNGC electrodes in a 0.05 M H_2_SO_4_ solution (pH ≃ 1). When the electrode has not been used, this erosion is negligible. Moreover, during the gelification step no significant volume contraction is appreciated what is very important for the mechanical stability of the electrodes. These results are consistent with literature [32]. In all cases, EDS spectra confirm the presence of Si, O and C: the first two ones corresponding to the Sonogel matrix and C as the massive modifier.
2. Concerning the CeO_2_-modified electrochemical sensors developed throughout this work, the CeO_2_ nanoparticles remain attached to the electrode surface in the form of a film even after many measurements (with more or less thickness according to the concentration of the nanomaterial) and without the presence of some protective agent or membrane (excepting for the CeO_2_(7.5 mg·mL^−1^)-modified electrode, which may have suffered from other problems, mainly during the drying step). However, with respect to the AuSNPs/CeO_2_-modified electrodes, the situation turns to be the opposite: most part of the film seems to fall down due to its weight, presence of ionic species coming from the AuSNPs synthesis solution and other unknown factors.
3. The presence of CeO_2_ or AuSNPs/CeO_2_ homogeneous film could be directly related to the electrochemical performances of the devices. Moreover, this might confirms that the electrocatalytic processes suffered by the different analytes take place preferentially on the nanoparticles surface.
4. Finally, the backscattered detector is a very powerful tool to help us to identify different phases according to the qualitative chemical composition of the electrode surface.

**Figure 9.**
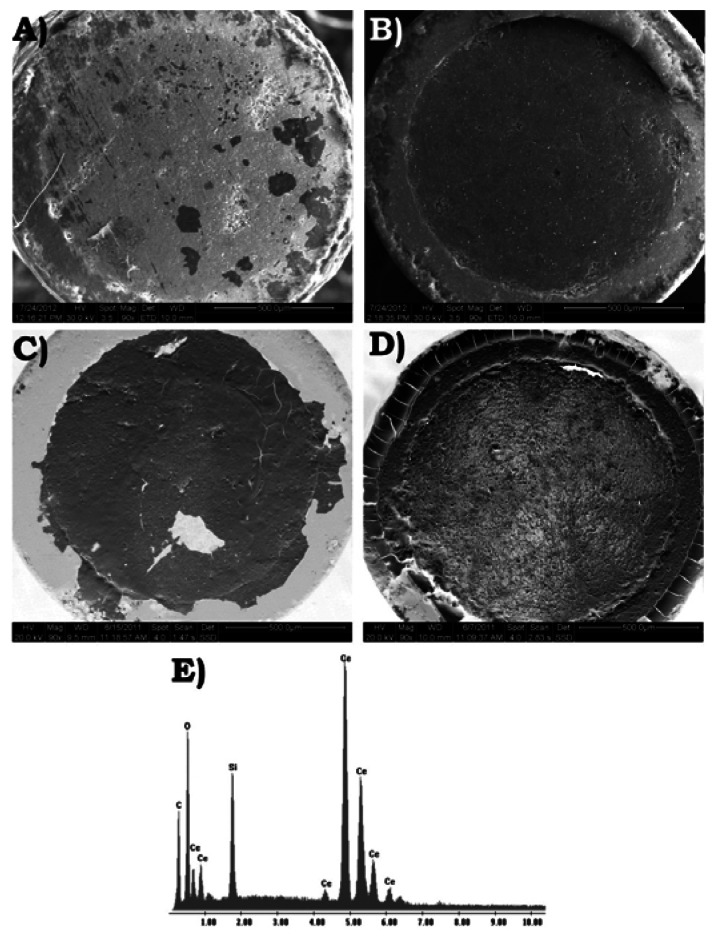
SEM micrographs and example of EDS corresponding to different configurations of the SNGC electrodes used and not used: CeO_2_(0.75 mg·mL^−1^)-modified SNGC electrode (**A**) used and (**B**) not used, both obtained with the secondary electron detector; CeO_2_(10.0 mg·mL^−1^)-modified SNGC electrode (**C**) used and (**D**) not used, both obtained with the backscattered electron detector; (**E**) X-ray EDS corresponding to the CeO_2_ nanoparticles film deposited on the surface of a SNGC electrode. All the micrographs were obtained at the magnification of 90× and operating in the range of 24-30 kV.

Figure 9 shows several SEM micrographs and example of EDS spectra corresponding to two different configurations of the CeO_2_-modified SNGC electrodes, used and not used. The micrographs correspond to the CeO_2_(0.75 mg·mL^−1^)- and CeO_2_(10.0 mg·mL^−1^)-modified SNGC electrodes, (A) used and (B) not used, for the first one, and (C) used and (D) not used, for the second one. As it can be seen, the surface of the SNGC electrodes is covered by a white film of CeO_2_ nanoparticles that appears in dark gray in micrographs (C) and (D), taken with a backscattered electron detector. When using this kind of detector it is very simple to identify different phases based on qualitative chemical composition of the electrode surface, especially when there is much difference in the atomic number, *i.e.*, SNGC matrix and glass of the capillary tube (light gray) and CeO_2_ nanoparticles. According to this, from Figure 9(C,D), CeO_2_ nanoparticle film can be distinguished from the SNGC matrix and information about how they coat the electrode surface can be extracted. In both devices, homogeneous film of cerium oxide nanoparticles can be observed, excepting for some small zones (dark gray in secondary electron detector and light gray in backscattered detector) corresponding to the Sonogel-Carbon matrix, although it could be assumed that small nanoparticles and not big aggregates still remain in these zones. However, it is obvious that the thickness of the film decrease when using the electrochemical device and that this fact might affect its performance. Nevertheless, from the results obtained, it is not possible to affirm categorically this relationship, mainly after testing that the electrocatalytic activity of these devices can be reproduced and repeated after many uses with most of the modified SNGC sensors, as we have seen previously from the CV and DPV studies. The fall can be also explained in terms of the mechanical fissures appearing on the film surface during the drying step, as observed in micrograph (D). The presence of CeO_2_ is corroborated by the EDS spectrum (Figure 9(E)), as well as, the components of the SNGC matrix: Si, C and O.

**Figure 10.**
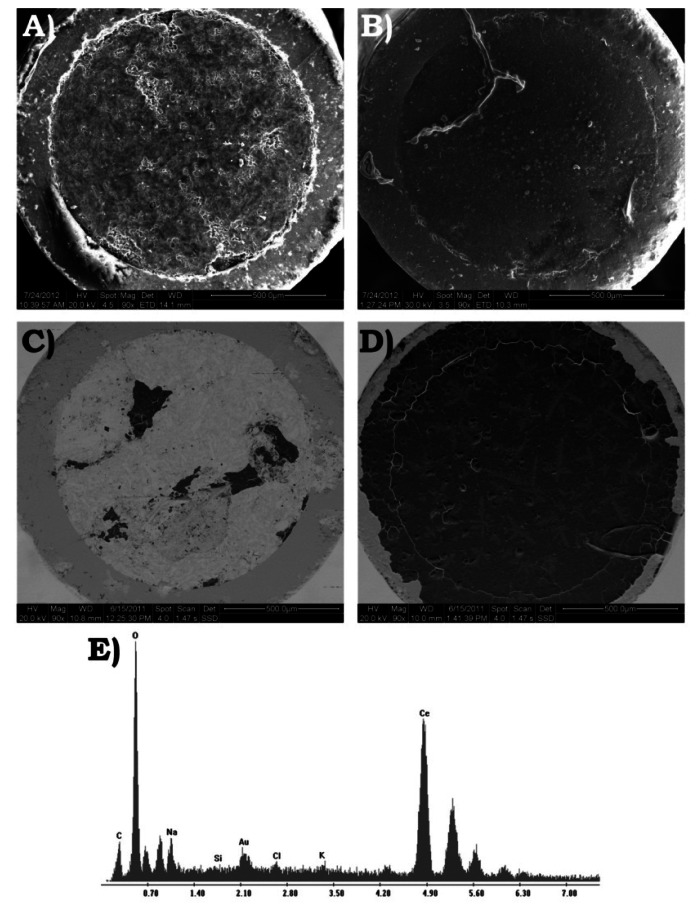
SEM micrographs and example of EDS corresponding to different configurations of the SNGC electrodes used and not used: AuSNPs/CeO_2_(25% w/w)-modified SNGC electrode (**A**) used and (**B**) not used, both obtained with the secondary electron detector; AuSNPs/CeO_2_(2.5% w/w)-modified SNGC electrode (**C**) used and (**D**) not used, both obtained with the backscattered electron detector; (**E**) X-ray EDS corresponding to the AuSNPs/CeO_2_ nanocomposite film deposited on the surface of a SNGC electrode. All the micrographs were obtained at the magnification of 90× and operating in the range of 24-30 kV.

Figure 10 shows the SEM micrographs and an example of the EDS spectra of two different configurations of the AuSNPs/CeO_2_-modified SNGC electrodes, used and not used. The micrographs correspond to the AuSNPs/CeO_2_(25% w/w)- and AuSNPs/CeO_2_(2.5% w/w)-modified SNGC electrodes, (A) used and (B) not used, for the first one, and (C) used and (D) not used, for the second one. As in the previous SEM micrographs, (C) and (D) were taken with a backscattered electron detector. From the figure, it can be concluded that the use of the electrochemical devices reduces significantly the AuSNPs/CeO_2_ film thickness in both electrodes, being more remarkable for higher proportions of AuSNPs/CeO_2_. Despite this, it seems that the nanocomposite film on the electrodes surface continues being homogeneous after their use.

As stated formerly, the AuSNPs/CeO_2_ film is heavier than the corresponding CeO_2_ film alone. Hence, the possibility of the film fall is higher in this case. Again, the EDS spectrum can be used to corroborate the presence of CeO_2_ and AuSNPs on the electrode surface (Figure 10(E)). The components of the SNGC matrix are also displayed (Si and C peaks are very small due to the EDS spot was focused on the film surface, with no much depth), and Na, K and Cl appear as new constituting elements. The origin of the last three ones can be attributed to the Au colloidal solution (K and Cl from KAuCl_4_, gold precursor, and Na from sodium citrate, reducing agent).

## 4. Conclusions

In this work, a complete study of the electrocatalytic activity of CeO_2_ nanoparticles and AuSNPs/CeO_2_ nanocomposite, deposited on the surface of the Sonogel-Carbon material used as supporting electrode, was accomplished. The modification of these electrochemical devices was done by simply drop-casting both nanomaterials on the electrode surface.

Different instrumental techniques were used to structurally characterize both the nanoparticles and the electrochemical devices built with them. These techniques provided us information about the bond of the CeO_2_ nanoparticles and the AuSNPs/CeO_2_ nanocomposite on the electrode surface: this bond seems to be strong enough to maintain them attached to the electrode surface, even after many measurements and without the presence of any protective agent or membrane.

CeO_2_- and AuSNPs/CeO_2_-modified SNGC electrodes were electrochemically characterized by CV and DPV. The electrocatalytic activity of AA, used as a benchmark analyte, was tested, obtaining in general good results. The main advantages given by the electrochemical devices described here can be summarized as follows: (i) AuNPs used here were synthesized by following a fast and green route and the procedure to “decorate” CeO_2_ nanoparticles with AuSNPs, only dependent of the stirring time, was very simple, this process being controlled by UV-vis spectroscopy; (ii) the final configuration of the sensing devices is much simpler and offers similar results, or even better in some cases, than most reported in literature; and (iii) it has also been demonstrated that most of the electrodes are rather sensitive, with a relatively high linear range of concentration, showing high reproducibility and repeatability when comparing with other AA sensors, even when part of the nanomaterial-based film is lost because of the continued use of the electrochemical sensors.

The electrochemical devices were also tested for the determination of AA in commercial apple juice for babies, obtaining comparable results with respect to the reference technique. Moreover, as far as we know, this is the second time that the electrochemical determination of AA in baby juices is reported (other methods have been used: enzymatic, chromatographic, but not electrochemical ones). This research suggests that the modified electrodes presented here can be a very promising voltammetric sensor for the determination of AA in real samples.

## Figures and Tables

**Table 1.** Experimental values of the observed capacity (*C*_obs_) at 100 mV·s^−1^ and the double-layer capacity (*C*_dl_) for the different configurations of the Sonogel-Carbon electrodes.

**Electrode**	C_obs_(μF/cm^2^) ± SD*	**C_dl_(μF/cm^2^) ± SD**
Bare SNGC	28 ± 1.76	24 ± 0.78
CeO_2_(0.25 mg·mL^−1^)-modified SNGC	46.82 ± 2.75	46.08 ± 4.49
CeO_2_(0.75 mg·mL^−1^)-modified SNGC	73.38 ± 8.87	72.44 ± 5.63
CeO_2_(1.0 mg·mL^−1^)-modified SNGC	105.83 ± 1.26	108.8 ± 8.56
CeO_2_(2.5 mg·mL^−1^)-modified SNGC	237.07 ± 6.97	228.14 ± 10.19
CeO_2_(5.0 mg·mL^−1^)-modified SNGC	243.24 ± 0.09	238.80 ± 3.96
AuSNPs/CeO_2_(25% w/w)-modified SNGC	99.86 ± 10.17	98.02 ± 10.68
AuSNPs/CeO_2_(12.5% w/w)-modified SNGC	97.96 ± 1.78	96.47 ± 3.56
AuSNPs/CeO_2_(5% w/w)-modified SNGC	44.97 ± 3.47	43.96 ± 0.65
AuSNPs/CeO_2_(2.5% w/w)-CeO_2_-modified SNGC	40.94 ± 10.35	41.93 ± 7.39

* SD: Standard Deviation.

**Table 2.** Results of the calibration curves obtained for AA when using the different types of Sonogel-Carbon electrodes employed.

**Electrode**	**Slope (×10^−3^A/M)**	**r^2^**
Bare SNGC	2.31	0.99991
CeO_2_(0.25 mg·mL^−1^)-modified SNGC	1.31	0.99845
CeO_2_(0.5 mg·mL^−1^)-modified SNGC	0.86	0.99763
CeO_2_(0.75 mg·mL^−1^)-modified SNGC	1.26	0.99996
CeO_2_(1.0 mg·mL^−1^)-modified SNGC	0.95	0.99910
CeO_2_(2.5 mg·mL^−1^)-modified SNGC	1.03	0.99966
CeO_2_(5.0 mg·mL^−1^)-modified SNGC	1.29	0.99990
CeO_2_(7.5 mg·mL^−1^)-modified SNGC	1.02	0.99994
CeO_2_(10.0 mg·mL^−1^)-modified SNGC	1.10	0.99832
AuSNPs/CeO_2_ (25% w/w)-modified SNGC	1.42	0.99995
AuSNPs/CeO_2_ (17.25% w/w)-modified SNGC	1.38	0.99969
AuSNPs/CeO_2_ (12.5% w/w)-modified SNGC	0.97	0.99900
AuSNPs/CeO_2_ (5% w/w)-modified SNGC	1.19	0.99974
AuSNPs/CeO_2_ (3.25% w/w)-CeO_2_-modified SNGC	1.31	0.99965
AuSNPs/CeO_2_ (2.5% w/w)-CeO_2_-modified SNGC	1.02	0.99940

**Table 3.** Results obtained for the reproducibility and repeatability studies by using three different SNGC electrodes and the same SNGC electrode (with at least three different calibration curves for each electrode), respectively, and the best detection and quantification limits for each one of the configurations tested.

	**Reproducibility Studies**		**Repeatability Studies**			

Electrode	Average slope (×10^−3^ A·M^−1^)	CV of slope (%)	Average slope (×10^−3^ A·M^−1^)	CV of slope (%)	LD (×10^−6^ M)	LQ (×10^−5^ M)
Bare SNGC	2.173	12.09	1.886	0.11	11.30	3.78
CeO_2_(0.25 mg·mL^−1^)-modified SNGC	1.015	9.77	1.072	0.67	0.55	1.83
CeO_2_(0.5 mg·mL^−1^)-modified SNGC	0.743	13.27	0.834	3.10	1.19	3.96
CeO_2_(0.75 mg·mL^−1^)-modified SNGC	1.054	21.71	1.236	1.96	0.16	0.52
CeO_2_(1.0 mg·mL^−1^)-modified SNGC	0.807	16.23	0.918	3.37	1.59	5.32
CeO_2_(2.5 mg·mL^−1^)-modified SNGC	0.991	2.71	0.995	3.19	2.51	8.36
CeO_2_(5.0 mg·mL^−1^)-modified SNGC	0.968	7.06	1.051	4.78	4.14	13.79
CeO_2_(7.5 mg·mL^−1^)-modified SNGC	0.935	7.67	0.943	7.17	3.88	12.94
CeO_2_(10.0 mg·mL^−1^)-modified SNGC	1.043	5.78	1.085	1.86	1.59	5.29
AuSNPs/CeO_2_(25% w/w)-modified SNGC	0.993	35.42	1.273	10.89	1.81	6.05
AuSNPs/CeO_2_(17.25% w/w)-modified SNGC	1.113	2.99	1.216	11.69	1.23	4.11
AuSNPs/CeO_2_(12.5% w/w)-modified SNGC	0.868	6.59	0.891	8.26	3.06	10.20
AuSNPs/CeO_2_(5% w/w)-modified SNGC	1.159	3.29	1.159	4.03	6.57	21.89
AuSNPs/CeO_2_(3.25% w/w)-CeO_2_-modified SNGC	1.223	6.24	1.285	2.48	2.93	9.77
AuSNPs/CeO_2_(2.5% w/w)-CeO_2_-modified SNGC	0.800	8.05	0.892	13.34	2.62	8.75

**Table 4.** Comparison of different CeO_2_−, AuNPs- and AuNPs/CeO_2_- modified electrodes for AA determination.

**Electrode**	**Method**	**LOD^1^(μM)**	**Linear Range (μM)**	**E (V)**	**Refs.**
βCD-nanoAu/Fc-ITO	CV	4.1	53–3,000	0.572	[46]
Functionalized-AuNPs/GCE	Amperometry	0.14	8–6,000	0.320	[47]
Au-PtNPs/Cys self-assembled/ITO	CV	1	2–400	0.250	[48]
AuNPs/PANI/GCE	Amperometry	0.5	3–20,000	0.300	[49]
AuNPs/silica/MWCNT/GCE	DPV	220	1,000–5,000	0.220	[50]
AuNPs/βCD/graphene/GCE	SWV	10	30–2,000	0.222	[51]
AuNPs/TiO_2_/Ti	DPV	400	1,000–5,000	0.350	[41]
AuSNPs/SNGC	DPV	3.71	1.5–4,000	0.104	[9]
MWCNT/GCE	SWV	1.4	4.7–5,000	0.315	[52]
PEDOT/Ni/Si/MCPE	DPV	10	20–1,400	0.030	[53]
SGN/NiPc	DPV	0.45	90–2,110	0.300	[54]
polyXa/MWCNT/GCE	Amperometry	0.1	1–1,520	0.300	[55]
CTAB/ABPE	2nd od LSV	1	2–1,000	0.284	[56]
GCE^2^	DPV	23.38	25–300	0.010	[57]
CeO_2_/GCE	DPV	1.50	5.0–1,000	0.092	[12]
PdNPs/CeO_2_/GCE	DPV	----	100–600	−0.083	[31]
CeO_2_/SNGC^3^	DPV	1.59	1.5–4,000	0.156	This work
AuSNPs/CeO_2_/SNGC^4^	DPV	2.93	1.5–4,000	0.140	This work

β-Cyclodextrin (βCD), Ferrocene (Fc), Cysteine (Cys), Polyaniline (PANI), Multi-walled carbon nanotubes (MWCNT), Sonogel-Carbon (SNGC), indium-tin-oxide (ITO), glassy-carbon electrode (GCE), poly(3,4-ethylenedioxythiophene) (PEDOT), microchannel plate electrode (MCPE), SiO_2_/C-graphite matrices (SGN), phthalocyanine (Pc), poly(xanthurenic acid) (polyXa), cetyl trimethyl ammonium bromide film (CTAB), acetylyne black paste electrode (ABPE), Square Wave Voltammetry (SWV), 2nd order derivative Linear Sweep Voltammetry (2nd od LSV;. ^1^ The limits of detection are based on S/N = 3 in all cases. ^2^ Electro-chemically treated GCE in basic medium (0.5 M NaOH). ^3^ Corresponding to the CeO_2_ (10.0 mg·mL^−1^)-modified SNGC electrode. ^4^ Corresponding to the AuSNPs/CeO_2_(3.25% w/w)-modified SNGC electrode.

**Table 5.** Experimental results for the determination of the content of AA in apple juice for babies by using the standard addition method.

**Electrode**	**Measured (mg·l^−1^)^1^± SD^2^**	**Measured (×10^−3^M) ± SD**	**Recovery (%) ± SD**
CeO_2_(0.25 mg·mL^−1^)-modified SNGC	232.2 ± 18.1	1.251 ± 0.05	101.56 ± 2.30
CeO_2_(0.5 mg·mL^−1^)-modified SNGC	220.4 ± 9.4	1.315 ± 0.07	106.22 ± 1.77
CeO_2_(0.75 mg·mL^−1^)-modified SNGC	231.6 ± 11.5	1.294 ± 0.04	104.54 ± 1.38
CeO_2_(1.0 mg·mL^−1^)-modified SNGC	227.9 ± 7.0	1.239 ± 0.05	100.07 ± 3.08
CeO_2_(2.5 mg·mL^−1^)-modified SNGC	218.2 ± 8.3	1.336 ± 0.02	107.95 ± 1.56
CeO_2_(5.0 mg·mL^−1^)-modified SNGC	235.3 ± 3.4	1.328 ± 0.01	107.3 ± 0.43
CeO_2_(7.5 mg·mL^−1^)-modified SNGC	233.9 ± 0.9	1.287 ± 0.03	103.99 ± 2.61
CeO_2_(10.0 mg·mL^−1^)-modified SNGC	226.7 ± 5.7	1.277 ± 0.01	103.17 ± 0.67
AuSNPs/CeO_2_(25% w/w)-modified SNGC	224.9 ± 1.5	1.242 ± 0.04	100.31 ± 2.63
AuSNPs/CeO_2_(17.25% w/w)-modified SNGC	218.7 ± 7.7	1.188 ± 0.04	96.01 ± 1.26
AuSNPs/CeO_2_(12.5% w/w)-modified SNGC	209.3 ± 7.1	1.225 ± 0.04	98.96 ± 2.08
AuSNPs/CeO_2_(5% w/w)-modified SNGC	215.7 ± 7.2	1.156 ± 0.03	93.41 ± 2.00
AuSNPs/CeO_2_(3.25% w/w)-CeO_2_-modified SNGC	203.6 ± 4.4	1.093 ± 0.003	88.76 ± 0.01
AuSNPs/CeO_2_(2.5% w/w)-CeO_2_-modified SNGC	193.5 ± 0.03	1.202 ± 0.03	97.09 ± 2.37

1 Reference value (HPLC): 1.24 × 10^−3^ M (218.0 mg·L^−1^); labelled average value: 1.42 × 10^−3^ M (250.0 mg·L^−1^).

2 SD = Standard deviation.
